# Evaluating the effectiveness of a radiation safety training intervention for oncology nurses: a pretest – intervention – posttest study

**DOI:** 10.1186/1472-6920-6-32

**Published:** 2006-06-08

**Authors:** Lawrence T Dauer, Joanne F Kelvin, Christopher L Horan, Jean St Germain

**Affiliations:** 1 Department of Medical Physics, Memorial Sloan-Kettering Cancer Center, 1275 York Ave. New York, NY 10021, USA; 2 Department of Radiation Oncology, Memorial Sloan-Kettering Cancer Center, 1275 York Ave. New York, NY 10021, USA

## Abstract

**Background:**

Radiation, for either diagnosis or treatment, is used extensively in the field of oncology. An understanding of oncology radiation safety principles and how to apply them in practice is critical for nursing practice. Misconceptions about radiation are common, resulting in undue fears and concerns that may negatively impact patient care. Effectively educating nurses to help overcome these misconceptions is a challenge. Historically, radiation safety training programs for oncology nurses have been compliance-based and behavioral in philosophy.

**Methods:**

A new radiation safety training initiative was developed for Memorial Sloan-Kettering Cancer Center (MSKCC) adapting elements of current adult education theories to address common misconceptions and to enhance knowledge. A research design for evaluating the revised training program was also developed to assess whether the revised training program resulted in a measurable and/or statistically significant change in the knowledge or attitudes of nurses toward working with radiation. An evaluation research design based on a conceptual framework for measuring knowledge and attitude was developed and implemented using a pretest-intervention-posttest approach for 15% of the study population of 750 inpatient registered oncology nurses.

**Results:**

As a result of the intervention program, there was a significant difference in nurse's cognitive knowledge as measured with the test instrument from pretest (58.9%) to posttest (71.6%). The evaluation also demonstrated that while positive nursing attitudes increased, the increase was significant for only 5 out of 9 of the areas evaluated.

**Conclusion:**

The training intervention was effective for increasing cognitive knowledge, but was less effective at improving overall attitudes. This evaluation provided insights into the effectiveness of training interventions on the radiation safety knowledge and attitude of oncology nurses.

## Background

Devine and Doyle[1] identified barriers to effective radiation therapy treatment for carcinoma that included staff's fears and misconceptions associated with radiation. Akers[2] points out that healthcare personnel, particularly those of childbearing age, are concerned about occupational exposures as they relate to fertility and pregnancy. These fears stem primarily from misconceptions and misunderstandings of radiation and the lack of knowledge of the effects of radiation[3]. Fear of radiation is highly communicable[2] and can negatively affect patient care. This concern is especially relevant to cancer patients who often search out or receive education about the risks and benefits of radiation treatments. Prior to entering the hospital, patients have generally come to accept the principle that the benefit of radiation treatment far outweighs the possible risks.

An understanding of radiation safety principles and their application in practice is critical for all oncology nurses. However, misconceptions about radiation are common, causing undue fears and concerns that may negatively impact patient care. Jankowski[4] has reported that nurses' fears about their exposure to radiation can be greatly reduced through education.

A multidisciplinary team of medical professionals including nurse leaders from radiation oncology and radiology, a nurse educator, clinical nurse specialists from inpatient units that commonly house patients admitted for radiation treatment, and radiation safety staff at MSKCC has developed a successful educational intervention addressing radiation safety knowledge and attitudes of nursing staff. A systematic evaluation of the efficacy of training methods for radiation safety education in oncology nursing has not been documented prior to this study.

**Figure 1:**
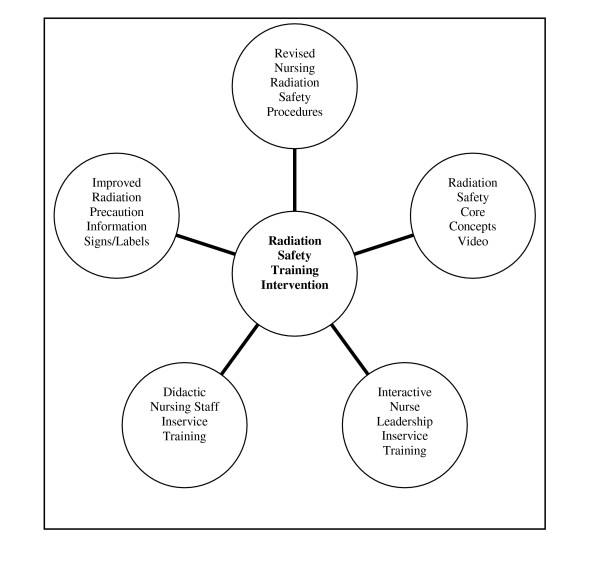
Elements of the MSKCC radiation safety training intervention for oncology nurses.

This study evaluated potential changes in nursing knowledge and attitudes with regard to the radiation safety program. The presumed cause of any possible differences in pretest versus posttest results was considered to be the training intervention[5,6] (Figure 1). Two different dependent variables were evaluated. The first was the cognitive knowledge that nurses display about the required radiation safety program. The second was the personal attitudes of nurses with regard to radiation and the radiation safety program at the hospital.

## Methods

### Design

Pretest-intervention-posttest designs are uniquely appropriate for investigating the effects of educational innovations[7] and are commonly used in educational research [8-10]. Strict experimental designs suggest the use of a two-group pretest-intervention-posttest design with a control group that receives no training intervention and a group that receives the training. In the occupational training environment required to meet regulatory compliance requirements, it is often not possible to allow differential services (i.e. different levels of training) for the staff. This was the case for radiation safety training at our cancer center where it was decided not to have a control group because the withholding of training from the control group would represent a differential service inconsistent with a worker's 'right to know' about the potential hazards. In this case, a one-group (no control) pretest-intervention-posttest experimental design was utilized. The absence of a control group was not considered a significant threat to the internal validity of the experiment because the likelihood that extraneous factors account for the change was small[5]. It was assumed that in the absence of the intervention (i.e. the specific radiation safety training programs) there were minimal or nonexistent outside variables that would have significantly changed a nurse's cognitive knowledge or attitude with regard to radiation safety regulations and policies over the pretest to posttest interval timeframes. It was concluded that the use of an experimental design without a control group was justified.

### Setting

The study was carried out in 2004 for inpatient staff registered nurses working with oncology patients at MSKCC, a National Cancer Institute designated comprehensive cancer center in New York City.

### Sample

All inpatient staff registered nurses (750 nurses) were considered as the study population. Participants were recruited by nurse educators and nurse leaders. A total of 15% of the registered nurses (i.e. 113 nurses) completed the study pretests and posttests. All nurses received the same training information.

### Intervention

The intervention consisted of a multifaceted set of improvements (Figure 1) including: nursing procedure revisions, a core concepts video, two types of inservice training, and enhanced "radiation precaution" signs and labels.

Radiation related nursing procedures were revised to maintain a consistent format and were edited to include only information that was considered to be essential information for nursing care situations. The procedures were validated by nursing and radiation safety experts for both scope and clarity.

A twelve-minute digital video was developed for incorporation into nursing orientation training and annual mandatory training for all nurses. Video topics were selected specifically to address the perceived knowledge gaps as well as to directly address fears of radiation. The video cast included a narrator, a professional male actor chosen by the nurse members of the multidisciplinary team, and representatives of the nursing and radiation safety staff community depicting various radiation safety precaution actions and discussions. An effort was made to include as many staff members as possible to heighten interest in the movie, help validate the material, and instill a sense of ownership. The video was digitized to facilitate presentation during training workshops as well as making the video available on the MSKCC intranet. Hyperlinks from the nursing procedures website allowed nurses to review the video at any time from virtually anywhere in the hospital.

Two types of inservice training sessions were held. The first was an interactive, hands-on workshop developed specifically for nursing leadership (managers, clinical nurse specialists, nurse practitioners, and nurse educators) with the goal of increasing their ability to problem solve in the area of radiation and radioactive precautions. The second was a didactic session for nursing staff on units that regularly housed patients requiring radioactive precautions. This inservice focused on the various radiation treatments and the associated precautions most likely to be experienced by nurses.

Highly visible "radiation precaution" door signs and chart labels were developed to specifically detail the required actions by nursing staff for maintaining the safety of patients and hospital staff while providing care for patients. The improved signs and labels better differentiated among the precautions required for permanent implants, temporary implants, and radiopharmaceutical therapy than the signs previously used.

### Instruments

Two instruments were designed for this evaluation. The first was designed to measure cognitive knowledge of radiation and radiation protection practices. The second was designed to measure attitudes of nurses with regard to radiation.

The present research relied upon a criteria-based multiple-choice/true-false test of cognitive knowledge [11-16]. The same instrument was utilized for both pretest and posttest evaluations. The cognitive test included 15 questions with 4 choices for each question. Face validity for the knowledge instrument was assessed by a local group radiation safety specialists and nursing leaders. Each question was scrutinized to ensure that it represented an accurate measure of desired parameters. Content validity was assessed through the use of radiation safety subject matter experts to ensure that proper topical coverage had been afforded by the overall test. [See additional file 1].

The cognitive questions addressed the following areas of knowledge: background radiation dose, annual limit, when to wear a badge, how to find exposure records, declared pregnant limit, radiosensitivity of the fetus, external beam treatment, seed implant treatment, visitor precautions, temporary implant treatment, permanent implant treatment, systemic radioiodine precautions, systemic radioiodine contamination, monitoring patient dose rate, and contamination cleanup protocols.

A Likert-scaled attitude evaluation included 9 questions. Face validity was assessed by a local group of radiation safety specialists and nursing leaders. [See additional file 1]. The instrument addressed the following attitudinal areas: I feel that radiation safety policies are clear, I know whom to contact for information, I know what steps to take, I can explain precautions well, I feel safe, policies are based on regulations, I am monitored, there is oversight, and I feel safe to have a child.

### Data analysis

Data were captured from the response sheets using the ReMark Office OMR software[17]. Two levels of data analysis were performed on the aggregate of pretest and posttest responses. The first used descriptive statistics and the second employed hypothesis evaluation statistics. The significance alpha level was chosen for all statistical tests to be 0.05, the most typical value for social research[6,18].

## Results

### Descriptive findings

Descriptive statistics including the number of tests graded, number of graded items, total score possible, maximum score, minimum score, median, mean, variance, and standard deviation were calculated for both the pretest and posttest cognitive scores (Table 1). The mean score on the cognitive test rose from 58.9% in the pretest to 71.2% in the posttest.

**Figure 2:**
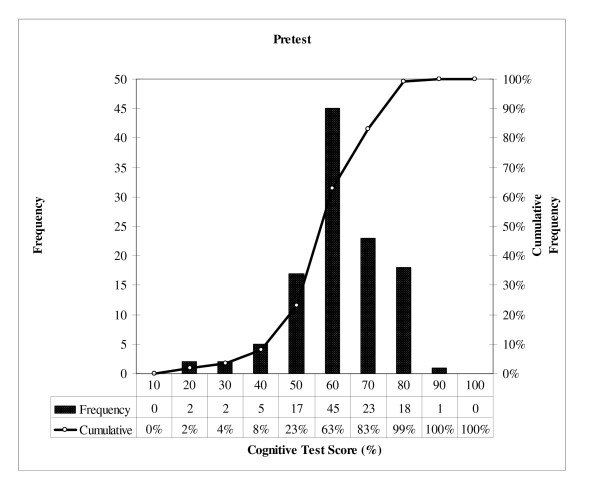
Frequency of scores for the cognitive test pretest scores.

**Figure 3:**
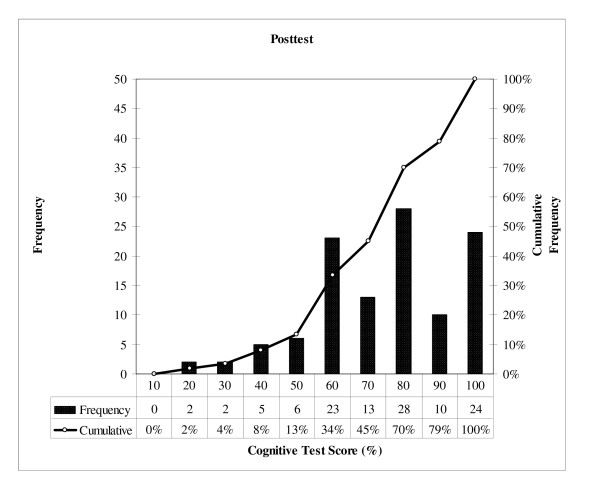
Frequency of scores for the cognitive test posttest scores.

The frequency distribution and the cumulative description of the cognitive test scores are shown in Figures 2 and 3. Figure 2 displays the results of the pretest which show a typical Gaussian result with scores centered around 60%. Figure 3 displays the results of the posttest and demonstrates scores skewed to the right (i.e. shifted toward higher scores).

**Figure 4:**
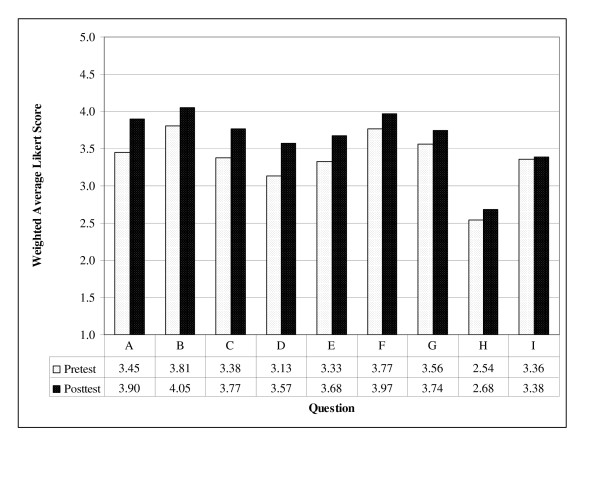
Weighted average Likert score pretest and posttest.

To assist in describing the attitude evaluation responses, a weighted average Likert score was calculated for the pretest and posttest attitude questions (Figure 4). For 8 out of 9 of the questions, the attitudes were generally positive, i.e. the weighted Likert score was greater than 3, for both the pretest and posttest responses. Responses to the question on oversight were generally negative, (i.e. the weighted Likert score was less than 3, for both the pretest and posttest. Figure 4 also shows that the weighted Likert score was higher in all cases for the posttest evaluation when compared to the lower scores in the pretest evaluation.

### Hypothesis evaluation findings

The t-test was utilized to test if as a result of the intervention program there would be no significant difference in nurse's cognitive knowledge as measured with the cognitive test instrument. Table 2 summarizes the results of the t-test analysis performed on the pretest and posttest data. The mean score for the 113 pretest results was 58.9% with a variance of 157.1. The mean score for the 113 posttest results was 71.6% with a variance of 385.7. The calculated t-value was 5.56, with a degree of freedom of 224 and a p = 1.85 × 10^-7^. This evaluation suggested that as a result of the intervention program, there was a significant difference in nurse's cognitive knowledge as measured with the instrument from pretest to posttest (i.e. nurses scored better on the posttest).

To evaluate the significance of differences on individual questions from pretest to posttest, t-test evaluations were performed on the results of each question. Table 3 lists the results of these t-tests. This evaluation suggested that as a result of the intervention program, there was a significant difference in nurse's cognitive knowledge on eight questions, as measured with the instrument from pretest to posttest. The evaluation also suggested that any differences observed in the remaining questions were not statistically significant.

A one-way chi-square test analysis for each of the attitude evaluation responses was performed and resulted in p-values that were all less than 0.05 (Table 4) demonstrating that the responses of each pretest and posttest attitude question differed significantly from chance and represented a measure of the attitudes of the nurses with regard to radiation safety.

Because the attitude evaluations were not the result of chance alone, the degree of difference between pretest attitudes and posttest attitudes was therefore evaluated. As observed in Figure 4, the posttest weighted Likert result was higher than the pretest weighted Likert result for all questions. The two-way chi-square test was used to evaluate the significance of these differences. Table 5 summarizes the results of the two-way chi-square tests. For each question, the table lists the calculated two-way chi-square test statistic, the degrees of freedom for each question, the p-value associated with these two-way chi-square test statistics, and whether or not the p-value was significant.

There was a significant relationship between the nurses' attitudes toward the radiation safety program and participation in the intervention program for the attitude survey questions concerning the following: I feel that radiation safety policies are clear, I know what steps to take, I can explain precautions well, I feel safe, and policies are based on regulations. Although the weighted average Likert score was higher in the posttests, the differences in pretest and posttest responses were not statistically significant for the following attitude survey questions: I know whom to contact for information, I am monitored, there is oversight, and I feel safe to have a child.

## Discussion

For the cognitive test, the pretest mean score was 58.9%; the posttest mean score was 71.6%; and the calculated t-value was significantly larger than the critical t-value. The evaluation suggested that as a result of the intervention program, there was a significant difference in nurses' cognitive knowledge.

The change in percent correct for each individual cognitive question from pretest to posttest (see Table 3) demonstrated that nurses scored better on thirteen of the fifteen questions. The changes were statistically significant for eight of these questions. Although the performance on the remaining seven questions did not change significantly, in almost all cases, greater than 75% of the nurses chose the correct answer on the pretest. This suggests that about 3 out of 4 nurses possessed a fundamental knowledge of these seven question areas prior to the intervention. In both the pretest and the posttest, less than half of the nurses correctly answered the question on the numerical annual limit of radiation exposure, suggesting that the intervention failed to significantly improve knowledge in this area.

Two-way chi-square test analyses of the attitude evaluation results demonstrated that there was a significant relationship between the radiation safety training intervention and the increase in agreement with 5 out of 9 of the attitude questions (Table 5). For the attitude evaluation survey, the Likert scale responses demonstrated that for 8 out of 9 of the questions, the attitudes were generally positive for both the pretest and posttest evaluations. The question on oversight (I feel that I will be called if I receive higher than normal exposures) was the only choice in which nurses generally disagreed with the statement. The weighted Likert score was higher, shifted to an improved, more agreeable attitude, in all cases for the posttest evaluation when compared to the lower scores in the pretest evaluation.

## Conclusion

Research on the outcomes of educational improvement interventions can be utilized to strengthen the theoretical basis for required regulatory training as well as to validate interventions. The radiation safety training intervention resulted in a statistically significant increase in the cognitive test scores. Therefore, it can be concluded that the elements of the training intervention successfully increased the general knowledge of radiation safety principles associated with oncology practices. It can also be concluded that while nursing knowledge levels significantly increased for more than half of the knowledge areas covered by the cognitive test, the measured knowledge levels did not comprehensively increase in all areas.

The pretest results of the attitude measures demonstrated that oncology nurses displayed a generally positive attitude with regard to radiation and radiation safety, even before the training intervention. Based on the data analysis of both the pretest and posttest attitude measures, nursing attitudes became more positive after the interventions. A statistically significant increase in survey responses was observed in 5 of the 9 of the questions. Therefore, it can be concluded that there was a relationship between the training intervention and the increase in attitude responses for some but not all of the areas.

When viewed together, the significant increase in cognitive knowledge and the mixed results of the attitude evaluation suggest that the training interventions were more successful at increasing knowledge and less successful at changing attitudes.

This study was limited by scope in both the specific nature of the training subject and the nature of the population. The study assumed that the choice of data gathering instruments was appropriate for the task at hand. As this study utilized voluntary participation rather than specific random sampling, extensions of these conclusions to other populations or individuals are understandably weakened.

### Recommendations for general practice

The training intervention has been incorporated into ongoing training programs. In addition, several strengths of the program can be adopted for use in other training program improvements. The involvement of stakeholders was essential for the development of the training intervention as well as the development and implementation of the research design. The use of a multidisciplinary team for the development of the training intervention elements resulted in high quality training tools that have been shown to be successful with the target audience.

The attitude evaluation results demonstrated that four areas (I know whom to contact for information, I am monitored, there is oversight, and I feel safe to have a child) should be emphasized in future radiation training intervention implementations. The training elements focused strongly on cognitive knowledge, with the assumption that an increase in knowledge would result in a concomitant improvement of attitudes. It may be possible to develop an additional training element that specifically addresses underlying assumptions and fears. Such an intervention might utilize open discussions or hands-on approaches. The addition of a behavioral psychologist to the multidisciplinary team may improve the outcome.

### Recommendations for future research

Further research is needed to focus on specifics. While this study evaluated the impact of the entire multi-element training intervention, no conclusions could be drawn for the individual elements of the intervention such as the video, the inservice training sessions, the policies and procedure, or the door signs. Which of these intervention elements had the most impact on increasing cognitive knowledge and/or attitudes is conjectural. The present study was not stratified by age, experience level, gender, hospital unit, or floor, to see if we could identify a nursing sub-population which is more difficult to educate.

Further research could broaden the study. While this study evaluated the performance and attitude of registered nurses on inpatient floors, a comparison of intervention results for other groups such as registered nurses versus nursing assistants may help to answer the question as to whether education level would affect outcomes. The study should also be extended to other practice settings.

While the present study utilized a specific set of attitude evaluation questions that concentrated on what the multidisciplinary team believed represented appropriate concerns of oncology nurses, all of the specific fears of the nurses were not requested or learned during this study. An expanded attitude evaluation survey and questionnaire could be beneficial in identifying real fears with respect to radiation. The intervention elements themselves could then be modified to include other methods designed to specifically address identified fears and/or misconceptions.

## Competing interests

The author(s) declare that they have no competing interests.

## Authors' contributions

LD conceived of the study, collected the bulk of the data, and drafted the manuscript. JK, CH, and JS participated in the design and coordination of the study. JS and JK revised the nursing procedures. All authors were members of the multidisciplinary team that designed the training intervention elements and test instrument. All authors also read and approved the final manuscript.

## Pre-publication history

The pre-publication history for this paper can be accessed here:



## Supplementary Material

Additional File 1 **Memorial Sloan-Kettering Cancer Center registered nurse radiation safety questionnaire**. This file contains the complete cognitive evaluation and attitude assessment questionnaire. The file is included as a standard pdf document. Click here for file

## Figures and Tables

**Table 1:** Summary of cognitive test result descriptive statistics

Descriptive Statistic	Pretest	Posttest
Number of Tests Graded	113	113
Number of Graded Items	15	15
Total Score Possible	100.0%	100.0%
Maximum Score	86.7%	100%
Minimum Score	20.0%	20.0%
Median Score	60.0%	73.3%
Mean Score	58.9%	71.2%
Variance	157.1	386.2
Standard Deviation	12.5	19.7

**Table 2:** t-test: paired two sample for means of the cognitive pretest versus posttest

	Pretest	Posttest	t-test
Number of Observations	113	113	
Mean	58.9%	71.6%	
Variance	157.1	385.7	
Degrees of freedom	112	112	224
Calculated t-value			5.56
Critical t-value (two-tail, alpha = 0.05)			1.98
p-value			1.85 × 10^-7^

**Table 3:** Change in percent correct for individual questions and t-test significance

Question	Pretest % Correct	Posttest % Correct	Change in % Correct	p-value	Significant? (p < 0.05)
Declared pregnant limit	13.4%	54.0%	40.6%	<0.0001	Y
Temporary implant treatment	15.3%	43.4%	28.0%	<0.0001	Y
Background radiation dose	48.2%	75.2%	27.0%	<0.0001	Y
Systemic radioiodine precautions	58.4%	80.4%	22.0%	0.0020	Y
Monitoring patient dose rate	61.6%	79.3%	17.7%	0.0033	Y
Permanent implant treatment	51.3%	65.8%	14.4%	0.0388	Y
Seed implant treatment	38.4%	52.7%	14.3%	0.0450	Y
Radiosensitivity of the fetus	53.1%	66.4%	13.3%	0.0351	Y
Annual limit	22.2%	30.1%	7.9%	0.1050	N
How to find exposure records	83.0%	89.4%	6.3%	0.0948	N
Visitor precautions	77.7%	83.0%	5.4%	0.3450	N
External beam treatment	87.5%	90.2%	2.7%	0.5509	N
Contamination cleanup protocols	91.2%	91.0%	-0.2%	0.6718	N
Systemic radioiodine contamination	91.2%	88.4%	-2.8%	0.3962	N
When to wear a badge	96.5%	92.0%	-4.4%	0.1665	N

**Table 4:** One-way chi-square test analyses for pretest and posttest attitude data

Question	Timeframe	Number of observations	Weighted average	One-way chi-square	*DF*	*p *value
I feel that policies are clear	Pretest	113	3.45	106.87	4	<0.00001
	Posttest	105	3.90	107.81	4	<0.00001
I know whom to contact	Pretest	113	3.81	134.92	4	<0.00001
	Posttest	105	4.05	144.10	4	<0.00001
I know what steps to take	Pretest	113	3.38	58.81	4	<0.00001
	Posttest	104	3.77	74.17	4	<0.00001
I can explain precautions well	Pretest	113	3.13	54.92	4	<0.00001
	Posttest	105	3.57	73.14	4	<0.00001
I feel safe	Pretest	113	3.33	66.25	4	<0.00001
	Posttest	105	3.68	84.67	4	<0.00001
Policies are based on regulations	Pretest	113	3.77	147.40	4	<0.00001
	Posttest	105	3.97	155.90	4	<0.00001
I am monitored	Pretest	111	3.56	102.47	4	<0.00001
	Posttest	104	3.74	86.29	4	<0.00001
There is oversight	Pretest	113	2.54	53.06	4	<0.00001
	Posttest	103	2.68	41.61	4	<0.00001
I feel safe to have a child	Pretest	108	3.36	61.91	4	<0.00001
	Posttest	99	3.38	66.81	4	<0.00001

**Table 5:** Two-way chi-square analyses based on pretest and posttest data

Question	Two-way chi-square	Degrees of freedom	p-value	Significant? (p < 0.05)
I feel that policies are clear	20.71	4	0.0004	Yes
I can explain precautions well	17.99	4	0.0012	Yes
I feel safe	12.28	4	0.0154	Yes
Policies are based on regulations	9.41	3	0.0243	Yes
I know what steps to take	10.65	4	0.0308	Yes
I know whom to contact	7.34	4	0.1188	No
There is oversight	4.26	4	0.3726	No
I am monitored	4.11	4	0.3915	No
I feel safe to have a child	0.71	4	0.9501	No
