# Supplementary material for: Evaluating the effectiveness of a radiation safety training intervention for oncology nurses: a pretest – intervention – posttest study
Source: BMC Med Educ. 2006 Jun 8;6:32. doi: 10.1186/1472-6920-6-32 (PMC1513562; doi:10.1186/1472-6920-6-32)
Supplement: Additional File 1 — Memorial Sloan-Kettering Cancer Center registered nurse radiation safety questionnaire. This file contains the complete cognitive evaluation and attitude assessment questionnaire. The file is included as a standard pdf document. [file 1472-6920-6-32-S1.pdf]

Additional File 1

Memorial Sloan-Kettering Cancer Center
Registered Nurse Radiation Safety Questionnaire

Cognitive Test:

Select the one correct response to each question. Where indicated, record your answers in the space provided.

1. What is the usual radiation exposure we receive each day?
   a. Zero. There is no normal background level of exposure.
   b. 10 millirem, equal to what is received from a chest x-ray.
   c. 2 millirem from cell phones and microwaves.
   d. 1 millirem from natural ever-present radiation.

2. What is the annual limit of radiation exposure for MSKCC staff as established by state and national regulatory agencies?
   A. 50 millirem per month.
   B. 100 millirem per year.
   C. 1,000 millirem per quarter.
   D. 5,000 millirem per year.

3. A person who has just been hired is issued a radiation monitoring badge. Which of the following statements is correct?
   A. If a person is exposed to radiation the badge will change color.
   B. The badge will provide some protection when working with patients on radiation precautions.
   C. The badge should be worn when providing care to a patient on radiation precautions.
   D. In case the badge is lost or misplaced, you can borrow a monitor from another staff member.

4. How can an employee who has been wearing a radiation monitoring badge find out how much radiation he or she has been exposed to over the last year?
   A. Read the total dose listed on the numbers on the back of the badge.
   B. Find their name and information on the reports that are sent to each nursing station or group leader.
   C. Call the Nuclear Regulatory Commission.
   D. Contact our radiation monitoring badge supplier.

5. A female employee has declared that she is pregnant and has received a special monitoring badge to wear while she is pregnant. Which of the following statements is correct?
   A. She will have to be re-assigned to areas where no radioactivity is used.
   B. Her total dose during pregnancy should be limited to 500 millirem.
   C. She will need to see her obstetrician more often than other pregnant women.
   D. The monitoring badge needs to be returned every two weeks.

6. Which of the following statements is correct with regard to a developing fetus?
   A. The level of risk from radiation exposure for the fetus is highest in the third trimester.
   B. A fetus exposed to low levels of radiation will have difficulty breast-feeding after birth.
   C. A staff member who has declared that she is pregnant should not be exposed to any radioactive patient during the first trimester.
   D. A developing fetus is more sensitive to radiation than an adult because cells are rapidly dividing.

7. Which of the following statements is correct with regard to external beam radiation treatment?
   A. When the patient returns to his or her room, the site being treated is still *slightly* radioactive.
   B. The more treatments the patient has, the more radioactive the patient becomes.
   C. Employees should avoid the patient’s room for 30 minutes after the patient returns.
   D. No radiation precautions are needed after the patient has been treated because the patient is not radioactive.

8. A patient under your care is receiving a temporary radioactive implant to treat a tumor located in his upper left thigh and the room has been posted with a yellow and magenta sign that states "Radioactive Precautions." All of the following statements are correct EXCEPT:
   A. Handle all items in the room as contaminated.
   B. Limit the amount of time that you spend in close contact with the patient.
   C. When possible, keep your distance from the patient when talking with him or her.
   D. Keep a rolling lead shield between you and the patient's thigh.

9. A patient under your care is receiving a temporary radioactive implant to treat cancer of the cervix. Which of the following statements is correct?
   A. Visiting times are restricted and visitors are only allowed to sit at the door to the room.
   B. There are no restrictions as long as the visitors are not pregnant.
   C. Visitors are allowed to stay at the bedside for a maximum of one hour.
   D. There are no restrictions as long as the visitors are over the age of 55.

10. A patient under your care is receiving temporary high dose rate brachytherapy (implant) to treat prostate cancer. Which of the following statements is correct?
   A. The patient needs a private room.
   B. The radiation treatment is administered in the patient’s room.
   C. The patient is not radioactive at any time while they are in their room on the inpatient unit.
   D. The patient is radioactive as long as the perineal catheters are in place.

11. A patient under your care recently received permanent radiation implants for prostate cancer. Which of the following statements is correct?
   A. Minimize the time you spend caring for this patient to reduce radiation exposure.
   B. The radiation does not penetrate much beyond the skin, so no special precautions are needed.
   C. Body fluids will be contaminated with radiation.
   D. Visitors should limit their time with the patient to one hour a day.

12. A thyroid cancer patient has been treated with systemic radioactive iodine. All of the following statements are correct EXCEPT:

A. The patient's door must remain closed at all times to stop radiation from escaping into the hallway.
B. All of the patient's linens and waste must remain in the room.
C. The patient must have a private room.
D. MSKCC employees may not enter the patient's room unless they are wearing a radiation monitoring badge.

13. A patient in your care is having treatment with systemic radioactive iodine for thyroid cancer. Shortly after administration of the treatment, the patient vomits. You find the vomitus on the floor and linens. Which of the following actions is correct?

A. Get the housekeeping staff to clean it up quickly before it spreads into the air.
B. Put on gloves, replace the linens yourself, and get housekeeping to clean up the floor.
C. Place a chuck over the affected area. Immediately call Radiation Safety to evaluate and decontaminate the room.
D. Ask the patient to step into the bathroom while you and the housekeeping staff clean up the room.

14. A patient in your care who has received a permanent radiation implant or treatment with systemic radioactive iodine is ready to be discharged. Which of the following radiation safety requirements must be met before the patient can leave the hospital?

A. Patients must no longer be emitting any radiation.
B. Patients must be monitored by Radiation Safety staff and have been given instructions on how to reduce exposure to family members.
C. No requirements are needed; once the patient is discharged by the physician, he or she can go home.
D. All the discharge requirements are posted on the radiation sign on the door.

15. After a patient who has received systemic radioactive iodine has gone home, what radiation safety requirements must be met before reusing the room?

A. There are no special radiation safety requirements.
B. Housekeeping must mop down the room at least 2 times with Clorox.
C. Radiation Safety staff need to be called to decontaminate the room and remove contaminated trash bins before housekeeping may enter the room.
D. The nurse should open the door to let the room air out before housekeeping may enter the room.

Attitude Evaluation:

For each of the following statements, select the response that most closely matches your opinion.

Strongly Disagree	Disagree	Neutral	Agree	Strongly Agree

A. The nursing policies and procedures on radiation precautions are clear and easy to understand.

B. I know whom to contact if I have questions about what radiation precautions are needed for a particular patient.

C. I feel confident about the steps I need to take when caring for patients needing radiation precautions.

D. I feel I can clearly explain the radiation precautions needed to my patients and their visitors.

E. I feel safe when caring for patients needing radiation precautions.

F. I feel the institutional policies and procedures are based on current regulations.

G. I feel confident the institution is carefully monitoring my radiation exposure.

H. I feel that I will be called if I receive higher than normal exposures.

I. I feel that working with patients receiving radiation will not affect my ability to have a child.
